# L-shaped association between triglyceride-glucose body mass index and short-term mortality in ICU patients with sepsis-associated acute kidney injury

**DOI:** 10.3389/fmed.2024.1500995

**Published:** 2024-12-06

**Authors:** Heping Xu, Ruiyong Mo, Yiqiao Liu, Huan Niu, Xiongwei Cai, Ping He

**Affiliations:** ^1^Department of Emergency Medicine, Hainan General Hospital/Hainan Affiliated Hospital of Hainan Medical University, Haikou, Hainan, China; ^2^Department of Emergency Medicine, Hainan Affiliated Hospital of Hainan Medical University, Haikou, Hainan, China

**Keywords:** triglyceride glucose-body mass index, acute kidney injury, sepsis, all-cause mortality, MIMIC-IV

## Abstract

**Background:**

Sepsis is a systemic inflammatory response syndrome, with sepsis-associated acute kidney injury (SA-AKI) being a common complication. Insulin resistance (IR) is closely related to the stress response, inflammatory response, and severity of critical illness. The triglyceride-glucose body mass index (TyG-BMI) is a valuable tool for assessing IR. However, the relationships between TyG-BMI and clinical outcomes in patients with SA-AKI remain unclear.

**Methods:**

We conducted a retrospective analysis of ICU patients with SA-AKI using data from the MIMIC-IV database. The Boruta algorithm was employed to select significant features for predicting short-term mortality in SA-AKI patients. Multivariate Cox proportional hazards regression, sensitivity analysis, restricted cubic spline (RCS) models, and Kaplan–Meier (K–M) survival analysis were used to assess the relationship between TyG-BMI and short-term mortality in SA-AKI patients. Subgroup analyses considered the effects of age, sex, ethnicity, comorbidities and septic shock.

**Results:**

This study included 3,349 patients, with males accounting for 60.5% of the patients. The Boruta analysis identified the TyG-BMI as an important clinical feature. Higher TyG-BMI values were significantly associated with reduced short-term mortality rates (28, 90, and 180 days) in patients with SA-AKI; for each standard deviation increase in TyG-BMI, the risk of all-cause death decreased by 0.2% (*p* < 0.0001). Kaplan–Meier analysis demonstrated that patients with high TyG-BMIs had significantly lower mortality rates than did those with low TyG-BMIs. The RCS model revealed an L-shaped nonlinear relationship between the TyG-BMI and mortality. Sensitivity analyses indicated that the association remained significant even after excluding patients with myocardial infarction, congestive heart failure, or those who were hospitalized in the ICU for less than 2 days. Subgroup analyses revealed a significant interaction effect on short-term mortality in CRRT patients (*p* < 0.05).

**Conclusion:**

The relationship between the TyG-BMI and short-term mortality in ICU patients with SA-AKI is significant, indicating its potential value for early risk assessment and clinical intervention.

## Introduction

Sepsis is defined as life-threatening organ dysfunction resulting from a dysregulated host response to infection (1). Despite significant advancements in understanding sepsis pathogenesis and developing clinical interventions, the incidence and mortality rates have shown only modest reductions (2, 3). Among the organs most severely impacted by sepsis are the kidneys, with studies indicating that 45–70% of acute kidney injury (AKI) cases are associated with this condition (4–7). Moreover, sepsis-associated acute kidney injury (SA-AKI) is closely linked to adverse clinical outcomes, such as prolonged hospital stays, increased cardiovascular events, and increased mortality rates (8, 9). Therefore, the early identification of patients at risk for SA-AKI, along with the timely implementation of appropriate interventions, is crucial for mitigating further renal damage.

The mechanisms underlying acute kidney injury induced by sepsis are multifaceted, and understanding these mechanisms is crucial for the timely identification and intervention of affected patients (9–11). First, sepsis is often associated with hypotension and organ hypoperfusion, which directly lead to renal tubular ischemia. Second, cytokines released during the systemic inflammatory response contribute to the damage of renal tubular cells and exacerbate inflammation, thereby intensifying kidney injury. Moreover, sepsis-induced immune dysfunction compromises the kidney’s protective mechanisms, further aggravating the development of AKI (5, 7, 9). In this context, identifying patients at risk for insulin resistance (IR) is particularly important for improving outcomes in septic individuals. The triglyceride-glucose (TyG) index has been proposed as a reliable marker for assessing IR, offering new insights into the clinical management and prognosis of patients with sepsis.

Insulin resistance, characterized by decreased sensitivity of peripheral tissues to insulin, is common among sepsis patients. However, researchers currently lack effective methods to assess IR precisely. The TyG index has emerged as a promising biomarker for evaluating IR (12). Given the strong association between IR and obesity (13), recent studies suggest that the TyG-BMI may serve as a valuable tool for assessing IR (14–16). Notably, however, the relationships between TyG-BMI and clinical outcomes in patients with SA-AKI have not been reported. This study aimed to investigate the association between TyG-BMI and the prognosis of patients with SA-AKI, thus addressing a significant gap in the literature.

## Methods

### Data source

The data for this study were obtained from the MIMIC-IV database (version 2.2) (17, 18), a publicly accessible registry developed by the Complex Systems Monitoring Group at Beth Israel Deaconess Medical Center (BIDMC) in Boston, Massachusetts. The dataset includes comprehensive records for over 50,000 patients admitted between 2008 and 2019, including demographic information, laboratory results, vital signs, disease diagnoses, and follow-up survival data. As the database is anonymized and lacks protected health information, the BIDMC Institutional Review Board approved a waiver of informed consent and permitted the use of the data for research purposes. Data extraction was performed by the corresponding author, Heping Xu, who completed the CITI Program online training course (Record ID 59568270) using PostgreSQL as the data management tool.

### Definitions

The TyG index was calculated as ln[(fasting glucose (mg/dl) × fasting TG (mg/dl))/2] (19). Body mass index (BMI) was calculated as body weight (kg)/height^2^ (m) (20). The TyG-BMI was computed according to the equation: TyG index × BMI (14). The TyG-BMI index was categorized into three tertiles: T1 (<235.7), T2 (235.7–294.8), and T3 (>294.8). Sepsis was diagnosed on the basis of the Sepsis-3 criteria, requiring an infection with a Sequential Organ Failure Assessment (SOFA) score of 2 or more. Septic shock was identified as sepsis accompanied by a lactate level exceeding 2.0 mmol/L and requiring vasopressor treatment (1). According to Kidney Disease: Improving Global Outcomes (KDIGO) guidelines, acute kidney injury was defined as an increase in serum creatinine (SCr) levels of ≥0.3 mg/dL from baseline within 48 h or a urine output of <0.5 mL/kg/h for 6 h (21). Baseline creatinine is defined as the lowest serum creatinine value within 7 days prior to ICU admission for AKI, which serves as the reference point for KDIGO staging. Sepsis-associated acute kidney injury is defined as the development of new-onset AKI within 7 days of ICU admission in patients with sepsis (22).

### Inclusion and exclusion criteria

#### Inclusion criteria

1. Patients aged 18 years or older.
2. Patients were diagnosed with sepsis-associated acute kidney injury.

#### Exclusion criteria

1. Patients with prior ICU admissions were excluded to prevent data duplication.
2. Patients whose survival time was less than 24 h were excluded to ensure sufficient evaluation of their clinical status and outcomes.
3. Patients with a history of chronic renal disease were excluded.
4. Patients missing essential data (serum fasting glucose, triglyceride, weight, height) or with incomplete data were excluded, as this information is critical for accurate calculation of the TyG-BMI.

### Outcome

The primary endpoint was 28-day all-cause mortality, while the secondary endpoints were all-cause mortality at 90 days and 180 days.

### Data extraction

The dataset extracted for this study included comprehensive demographic and clinical variables, such as age, sex, race, weight, height, history of myocardial infarction, congestive heart failure, chronic pulmonary disease, diabetes, and cerebrovascular disease. It also encompasses the initial SOFA score, the Simplified Acute Physiology Score II (SAPS II), and the Charlson Comorbidity Index. The recorded vital signs included systolic and diastolic blood pressure, mean arterial pressure, heart rate, respiratory rate, temperature, and pulse oximetry readings. The laboratory parameters included white blood cell count, hemoglobin, platelet count, anion gap, bicarbonate, chloride, glucose, triglycerides, sodium, potassium, creatinine, blood urea nitrogen, calcium, and prothrombin time. The monitored clinical outcomes included septic shock, CRRT, invasive ventilation, AKI, hospital mortality, and mortality rates at 28 days, 90 days, and 180 days. Additionally, the ICU length of stay and total hospital stay were recorded. All baseline data were collected within 24 h prior to ICU admission to ensure accuracy.

### Statistical analysis

In this study, continuous variables are presented as the means (standard deviations) or medians (interquartile ranges), whereas categorical variables are expressed as percentages. Baseline characteristics across different TyG-BMI categories were assessed via the chi-square test for categorical data, one-way ANOVA for normally distributed continuous data, and the Kruskal–Wallis H test for nonnormally distributed continuous data.

To identify important features predictive of short-term mortality in SA-AKI patients, the Boruta algorithm was used to evaluate the significance of the TyG-BMI as a predictor. This algorithm determines feature importance by comparing the Z value of each real feature to the maximum Z value of the corresponding “shadow feature.” A feature is marked as “important” (green area) if its Z value is significantly higher than the maximum Z value of the shadow features across multiple independent tests; otherwise, it is marked as “unimportant” (red area) and excluded from the feature selection process. The Boruta algorithm’s default parameters included a significance level of *p* = 0.01 and a maximum of 100 iterations (23).

To investigate the relationship between the TyG-BMI and all-cause short-term mortality in SA-AKI patients, multivariable Cox proportional hazards regression analysis was conducted. The Boruta algorithm identified 31 important features, including the TyG-BMI. Four models were developed, each progressively adjusted: Model 1 was the unadjusted baseline model; Model 2 adjusted for age, ethnicity, cerebrovascular disease, Charlson Comorbidity Index, SOFA score, SAPS II score, invasive ventilation, CRRT, and shock-related variables; Model 3 further adjusted for systolic blood pressure, diastolic blood pressure, mean arterial pressure, heart rate, respiratory rate, temperature, and SpO2; Model 4 additionally adjusted for white blood cell count, hemoglobin, platelet count, anion gap, bicarbonate, sodium, potassium, creatinine, blood urea nitrogen, calcium, chloride, prothrombin time, glucose, and triglycerides on the basis of Model 3.

Subgroup analyses were performed on the basis of age (<65 years and ≥ 65 years), sex, race, history of myocardial infarction, congestive heart failure, cerebrovascular disease, chronic pulmonary disease, diabetes, CRRT and septic shock. Sensitivity analyses included Cox proportional hazards regression analyses excluding patients with myocardial infarction and those with both myocardial infarction and congestive heart failure to further validate the results. Analyses were also conducted by excluding patients with an ICU stay of less than 2 days to ensure the robustness of the findings.

To determine the nonlinear relationship between the TyG-BMI and short-term mortality in SA-AKI patients, restricted cubic spline curves were plotted for visualization. Kaplan–Meier survival analysis was used to compare survival rates among ICU patients with SA-AKI stratified by TyG-BMI and to assess the impact of TyG-BMI on short-term mortality in SA-AKI patients. All data analyses were performed via R version 4.2.1 and Stata version 18.0. Missing values (<2%) were imputed using the median. Statistical tests were two-sided, and a *p*-value of less than 0.05 was considered statistically significant.

## Results

### Baseline characteristics of the participants

**Figure 1:**
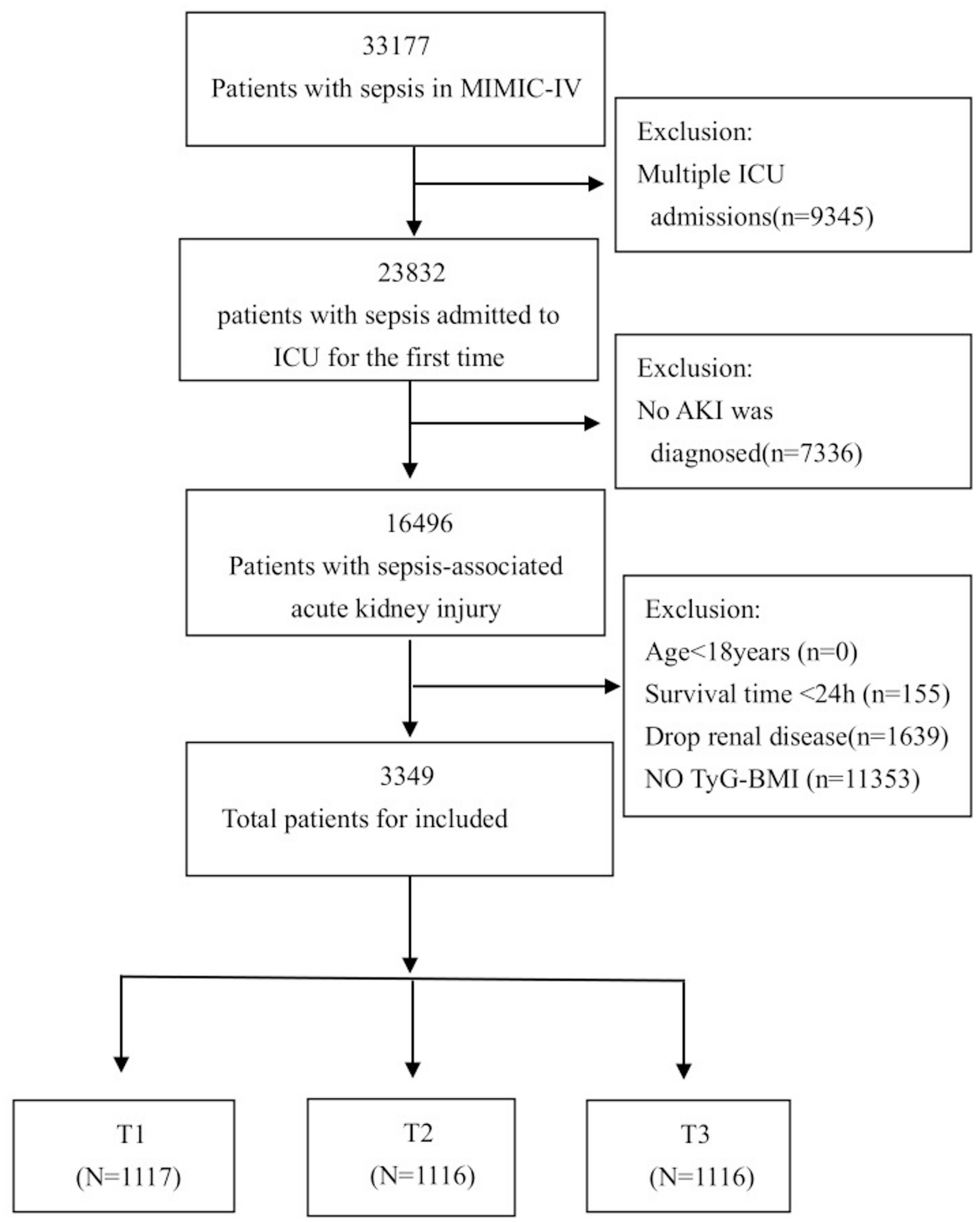
Flow chart of patient selection for analysis.

In this study, a total of 3,349 patients met the inclusion criteria, with 11,353 patients excluded due to missing TyG-BMI data, as shown in Figure 1. Table 1 presents a comprehensive summary of the baseline characteristics of these patients, stratified by TyG-BMI. The mean age was 64.1 years (SD = 15.5), with approximately 60.5% of the participants being male. The TyG-BMI was categorized into three tertiles: T1 (<235.7), T2 (235.7–294.8), and T3 (>294.8).

**Table 1:** Baseline characteristics and outcomes of patients with sepsis-associated acute kidney injury stratified by TyG-BMI.

Characteristics	TyG-BMI				
	Total (*N* = 3,349)	T1 (*N* = 1,117)	T2 (*N* = 1,116)	T3 (*N* = 1,116)	*p*
TyG-BMI	277.6 (83.5)	201.3 (25.5)	263.2 (17.1)	368.5 (75.3)	<0.0001
Demographic					
Age, years	64.1 (15.5)	67.3 (16.4)	64.3 (15.3)	60.6 (13.9)	<0.0001
Sex (male, *n*)	2025 (60.5)	672 (60.2)	734 (65.8)	619 (55.5)	<0.0001
Ethnicity (white, *n*)	2,233 (66.7)	745 (66.7)	774 (69.4)	714 (64.0)	0.027
Comorbidities					
Myocardial infarction	557 (16.6)	192 (17.2)	192 (17.2)	173 (15.5)	0.463
Congestive heart failure	735 (21.9)	245 (21.9)	236 (21.1)	254 (22.8)	0.655
Chronic pulmonary disease	797 (23.8)	264 (23.6)	221 (19.8)	312 (28.0)	<0.0001
Diabetes	881 (26.3)	194 (17.4)	262 (23.5)	425 (38.1)	<0.0001
Cerebrovascular disease	548 (16.4)	201 (18.0)	181 (16.2)	166 (14.9)	0.136
Severity scores					
Charlson comorbidity index	5 (3–6)	5 (4–7)	5 (3–6)	5 (3–6)	0.0003
First day of SOFA	7 (5–10)	7 (4–10)	7 (4–10)	8 (5–11)	<0.0001
SAPSII	40 (31–50)	40 (32–50)	39 (31–50)	40 (32–50)	0.112
Vital signs					
SBP, mmHg	112.0 (104.5–122.0)	111.2 (103.1–121.0)	112.0 (104.9–122.1)	112.8 (105.1–123.4)	0.003
DBP, mmHg	60.7 (55.1–67.3)	60.3 (54.7–657.0)	60.7 (55.0–67.2)	61.1 (55.6–67.8)	0.032
MBP, mmHg	75.7 (70.4–82.3)	74.9 (69.7–81.1)	75.8 (70.6–82.7)	76.1 (70.8–82.8)	0.0016
Heart rate, beats/min	86.3 (76.6–98.6)	85.1 (75.4–97.3)	85.5 (76.3–97.1)	88.2 (78.2–101.2)	<0.0001
Respiratory rate, beats/min	19.3 (16.9–22.5)	19.1 (16.6–22.4)	18.7 (16.7–21.7)	20.1 (17.6–23.5)	<0.0001
Temperature, °C	36.9 (36.6–37.3)	36.9 (36.6–37.2)	36.9 (36.6–37.2)	36.9 (36.7–37.4)	<0.0001
SpO2, %	97.3 (95.8–98.6)	97.6 (96.0–98.9)	97.5 (96.0–98.7)	97.1 (95.5–98.3)	<0.0001
Laboratory parameters					
WBC, cell/mm3	12.4 (9.2–16.3)	11.8 (8.8–15.6)	12.3 (9.0–16.0)	13.0 (9.7–17.2)	<0.0001
Hemoglobin, mg/dL	10.6 (9.3–12.2)	10.5 (9.1–12.1)	10.6 (9.3–12.1)	10.9 (9.4–12.4)	0.0003
Platelet, cell/mm3	177.5 (127.5–241)	180.5 (130–251.5)	170.5 (119–232.2)	180.5 (133.5–243.8)	0.004
Anion gap, mEq/L	14.0 (12.0–16.5)	14.0 (12.0–16.5)	14 (12.0–16.2)	14.5 (12.0–17.5)	0.0004
Bicarbonate, mEq/L	22.5 (20.0–25.0)	22.5 (20.0–25.0)	22.5 (20.0–25.0)	22.5 (19.8–25.0)	0.959
Chloride, mEq/L	105.0 (101.0–108.0)	105.0 (101.0–108.0)	105 (102–108.5)	104.5 (100.8–107.5)	0.0003
Sodium, mEq/L	138.5 (136.0–141.0)	138.5 (136.0–141.0)	138.5 (136–141)	138.5 (136.0–141.0)	0.624
Potassium, mEq/L	4.2 (3.85–4.6)	4.15 (3.8–4.5)	4.2 (3.85–4.58)	4.25 (3.9–4.65)	<0.0001
Calcium, mg/dL	8.25 (7.8–8.6)	8.25 (7.8–8.6)	8.25 (7.8–8.6)	8.25 (7.8–8.6)	0.505
Creatinine, mg/dL	1.0 (0.75–1.4)	0.9 (0.7–1.3)	0.95 (0.75–1.35)	1.05 (0.8–1.6)	<0.0001
BUN, mg/dL	19.0 (14.0–29)	19.0 (13.0–30.0)	18.5 (13.5–27.0)	20.0 (14.5–31.0)	0.0004
PT, sec	14.6 (13.0–17.3)	14.6 (13.0–17.3)	14.5 (13.1–17.3)	14.5 (13.0–17.1)	0.258
Glucose, mg/dL	133.5 (116.4–161.6)	125.5 (107.7–145.7)	132.6 (118.2–159.2)	145.2 (124.7–182.5)	<0.0001
Triglycerides, mg/dL	126 (86–189)	96 (69–137)	127 (88–182)	171 (116–267.5)	<0.0001
Outcome					
CRRT	383 (11.4)	79 (7.1)	124 (11.1)	180 (16.1)	<0.0001
Invasive ventilation	1,373 (41.0)	429 (38.4)	456 (40.9)	488 (43.7)	0.038
Septic shock	2,132 (63.7)	682 (61.1)	725 (65.0)	725 (65.0)	0.086
LOS ICU	5.3 (2.6–11.1)	5.2 (2.7–10.0)	5.1 (2.4–10.8)	5.9 (2.6–12.8)	0.008
LOS hospital	11.8 (6.9–19.9)	11.7 (6.9–19.6)	11.7 (6.7–19.7)	12.3 (6.9–20.8)	0.304
In-hospital mortality	708 (21.1)	271 (24.3)	206 (18.5)	231 (20.7)	0.003
28-day mortality	726 (21.7)	300 (26.9)	202 (18.1)	224 (20.1)	<0.0001
90-day mortality	938 (28.0)	383 (34.3)	272 (24.4)	283 (25.4)	<0.0001
180-day mortality	1,027 (30.7)	424 (38.0)	299 (26.8)	304 (27.2)	<0.0001

Continuous variables are presented as the means (SDs) or medians (quartiles), whereas categorical variables are presented as absolute numbers (percentages). SBP, systolic blood pressure; DBP, diastolic blood pressure; MBP, mean blood pressure; SpO2, pulse oxygen saturation; TyG, triglyceride–glucose; SOFA, Sequential Organ Failure Assessment; SAPS II, simplified acute physiology score II; WBC, white blood cell; BUN, blood urea nitrogen; PT, prothrombin time; CRRT, continuous renal replacement therapy; LOS, length of stay; TyG-BMI, triglyceride glucose-body mass index.

### Boruta algorithm

**Figure 2:**
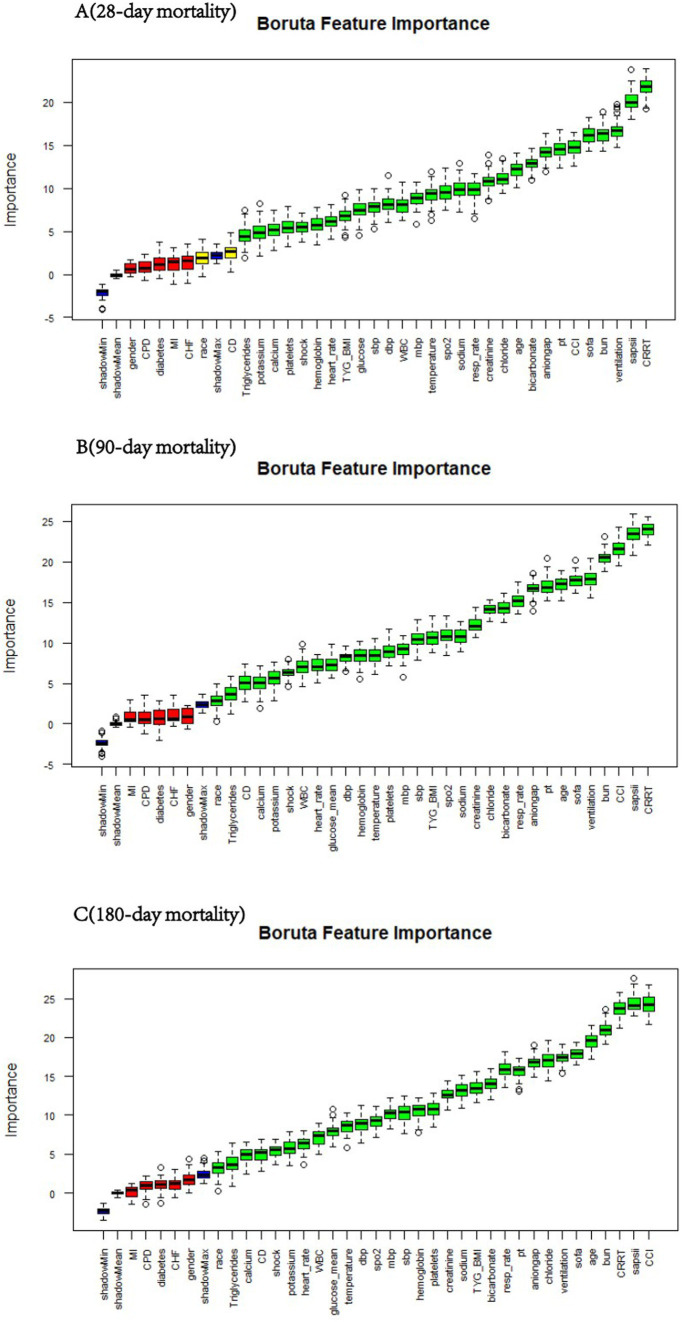
Feature selection for predicting short-term mortality risk via the Boruta algorithm. The horizontal axis represents the name of each variable, and the vertical axis represents the Z value of each variable. The box plot shows the Z value of each variable during model calculation. The green boxes represent important variables, and the red boxes represent unimportant variables. SBP, systolic blood pressure; DBP, diastolic blood pressure; MBP, mean blood pressure; SpO2, pulse oxygen saturation; MI, myocardial infarction; CHF, congestive heart failure; CD, cerebrovascular disease; CPD, chronic pulmonary disease; CRRT, continuous renal replacement therapy; CCI, Charlson comorbidity index. TyG, triglyceride–glucose; TyG-BMI, TyG × BMI; SOFA, Sequential Organ Failure Assessment score; SAPS II, simplified acute physiology score II; WBC, white blood cell; BUN, blood urea nitrogen; PT, prothrombin time.

We employed the Boruta algorithm to identify features associated with short-term mortality in patients with sepsis-associated acute kidney injury, as shown in Figure 2. In the Boruta analysis, variables in the green zone were classified as important features, whereas those in the red zone were deemed nonessential. The results indicate that the TyG-BMI was consistently identified as a significant predictive factor for mortality risk at 28, 90, and 180 days through the Boruta algorithm.

### Associations of the TyG-BMI with the clinical outcomes of SA-AKI patients

The relationships between the TyG-BMI and clinical outcomes are detailed in Table 2. Patients were categorized into three groups on the basis of TyG-BMI. We utilized four different Cox proportional hazards regression models to assess the independent effect of the TyG-BMI on mortality in ICU patients with sepsis-related acute kidney injury. Cox proportional hazards regression analysis revealed a negative correlation between the TyG-BMI and mortality risk at 28, 90, and 180 days. After all the clinical covariates were adjusted, each unit increase in the TyG-BMI was associated with a reduction in the risk of all-cause death decreased by 0.2% at 28, 90 and 180 days, respectively (*p* < 0.0001). Using the lowest TyG-BMI group as a reference, the analysis of TyG-BMI as a categorical variable (by quartile) revealed that mortality rates at 28, 90, and 180 days decreased with increasing TyG-BMI (*p* < 0.05).

**Table 2:** Association between the TyG-BMI index and all-cause mortality in SA-AKI according to different models.

TyG-BMI	Model1 HR (95% CI) *p* value		Model2 HR (95% CI) *p* value		Model3 HR (95% CI) *p* value		Model4 HR (95% CI) *p* value	
28-day mortality	0.999 (0.998,0.999)	0.003	0.998 (0.997,0.999)	<0.0001	0.998 (0.997,0.999)	<0.0001	0.998 (0.997,0.999)	0.001
Tertile1	Ref		Ref		Ref		Ref	
Tertile2	0.651 (0.544,0.778)	<0.0001	0.625 (0.521,0.748)	<0.0001	0.656 (0.547,0.787)	<0.0001	0.679 (0.564,0.818)	<0.0001
Tertile3	0.729 (0.613,0.867)	<0.0001	0.657 (0.549,0.785)	<0.0001	0.641 (0.535,0.770)	<0.0001	0.687 (0.561,0.840)	<0.0001
*p* for trend	<0.0001		<0.0001		<0.0001		<0.0001	
90-day mortality	0.998 (0.998,0.999)	<0.0001	0.998 (0.997,0.999)	<0.0001	0.998 (0.997,0.999)	<0.0001	0.998 (0.997,0.999)	<0.0001
Tertile1	Ref		Ref		Ref		Ref	
Tertile2	0.673 (0.576,0.786)	<0.0001	0.653 (0.558,0.764)	<0.0001	0.695 (0.593,0.814)	<0.0001	0.714 (0.607,0.840)	<0.0001
Tertile3	0.708 (0.607,0.8262)	<0.0001	0.648 (0.553,0.759)	<0.0001	0.636 (0.541,0.747)	<0.0001	0.683 (0.572,0.816)	<0.0001
*p* for trend	<0.0001		<0.0001		<0.0001		<0.0001	
180-day mortality	0.998 (0.997,0.999)	<0.0001	0.998 (0.997,0.999)	<0.0001	0.998 (0.997,0.999)	<0.0001	0.998 (0.997,0.999)	<0.0001
Tertile1	Ref		Ref		Ref		Ref	
Tertile2	0.662 (0.571,0.768)	<0.0001	0.650 (0.559,0.755)	<0.0001	0.693 (0.596,0.805)	<0.0001	0.712 (0.610,0.831)	<0.0001
Tertile3	0.681 (0.588,0.789)	<0.0001	0.632 (0.543,0.736)	<0.0001	0.623 (0.534,0.726)	<0.0001	0.673 (0.568,0.797)	<0.0001
*p* for trend	<0.0001		<0.0001		<0.0001		<0.0001	

HR, hazard ratio; CI, confidence interval; Model 1: Unadjusted model. Model 2: Adjusted for age, ethnicity, cerebrovascular disease, the Charlson comorbidity index, SOFA score, SAPSII score, septic shock, invasive ventilation and CRRT. Model 3: Adjusted for variables included in Model 2 + SBP, DBP, MAP, heart rate, respiratory rate, temperature and SpO2. Model 4: Adjusted for variables included in Model 3 + white blood cell count, hemoglobin, platelet count, anion gap, bicarbonate, calcium, blood urea nitrogen, sodium, potassium, chloride, creatinine, prothrombin time, glucose and Triglycerides.

Additionally, we conducted further Cox regression analyses to assess the impact of including BMI and TyG as covariates in four models (Supplementary Tables S1, S2). When BMI was included as a covariate, the hazard ratios (HR) for 28-day, 90-day, and 180-day mortality decreased to 0.990 (95% CI: 0.982–0.998, *p* = 0.019), 0.994 (95% CI: 0.986–0.999, *p* = 0.002), and 0.993 (95% CI: 0.986–1.000, *p* = 0.024), respectively. In contrast, when TyG was included as a covariate, the HRs for 28-day, 90-day, and 180-day mortality remained largely unchanged. This suggests that the effect of TyG-BMI on mortality in ICU patients with sepsis-associated AKI may primarily be driven by BMI, rather than TyG alone.

### Restricted cubic spline

**Figure 3:**
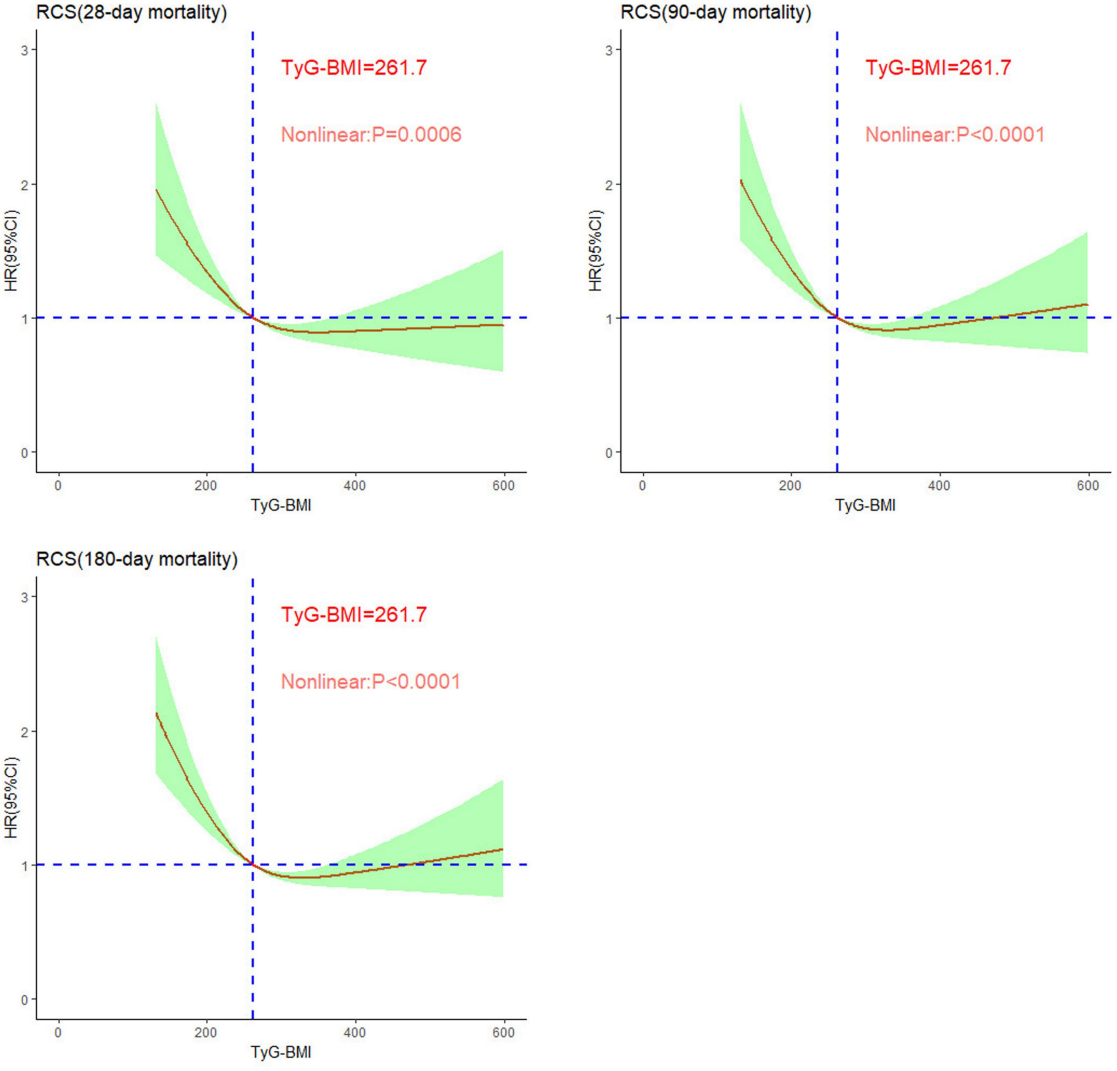
Nonlinear relationships between TyG-BMI and short-term mortality rates in patients with sepsis-associated acute kidney injury.

We established the threshold for the TyG-BMI via restricted cubic splines (RCSs) to illustrate the nonlinear relationship between the TyG-BMI at ICU admission and mortality at 28, 90, and 180 days. As shown in Figure 3, the TyG-BMI demonstrated a nonlinear correlation with mortality risk in patients with SA-AKI (*p* < 0.001), following an L-shaped curve. Specifically, when the TyG-BMI was less than 261.7, the risk of mortality at 28, 90, and 180 days increased sharply as the TyG-BMI decreased. Conversely, When the TyG-BMI exceeds 261.7, its impact on short-term mortality no longer shows significant variation. Overall, higher TyG-BMI values at ICU admission are associated with reduced short-term mortality.

### Kaplan–Meier analysis

**Figure 4:**
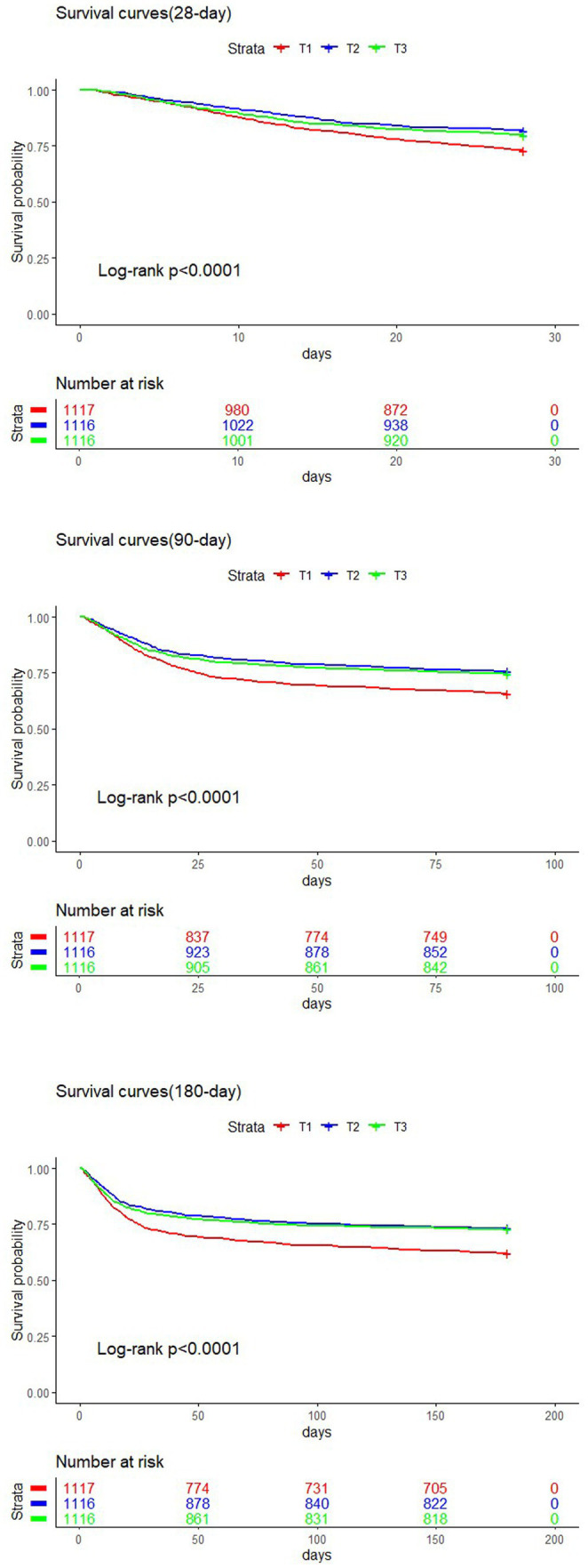
Kaplan–Meier plots for short-term mortality by ICU admission TyG-BMI strata.

The study population was divided into three groups based on TyG-BMI tertiles: T1, T2, and T3. Kaplan–Meier survival analysis was conducted to evaluate short-term mortality rates in patients with SA-AKI. As illustrated in Figure 4, the survival curves for the T1 groups were significantly lower than those for the T2 and T3 group (log-rank test, *p* < 0.0001). No statistically significant difference was observed between the T2 and T3 groups (*p* > 0.05), which indicates that a lower TyG-BMI at admission is associated with higher short-term mortality rates.

### Subgroup analysis

**Figure 5:**
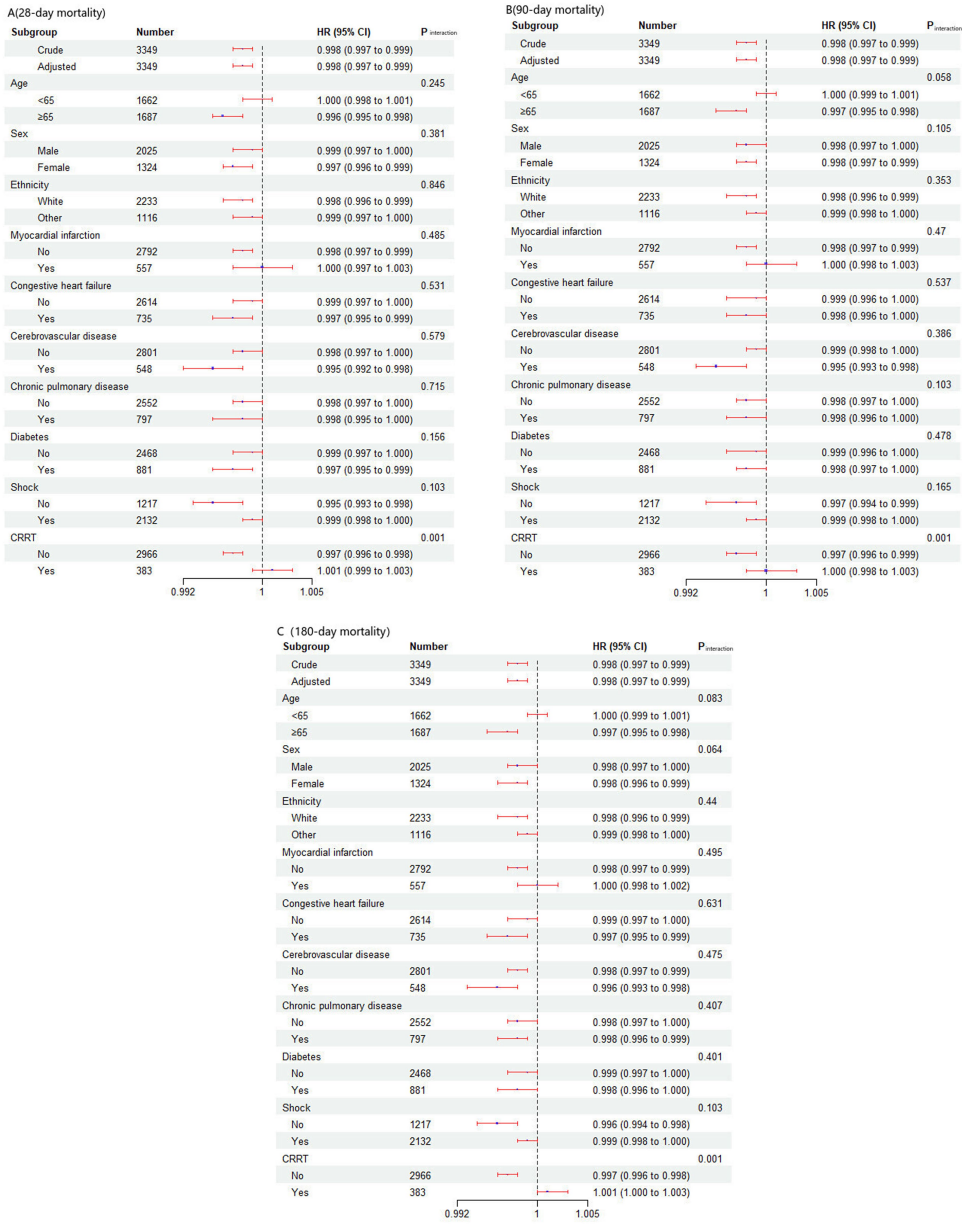
Effect size of the TyG-BMI on short-term mortality in prespecified and exploratory subgroups. The effect size was adjusted for age, ethnicity, cerebrovascular disease, Charlson comorbidity index, SOFA score, SAPSII score, Septic shock, invasive ventilation, CRRT, SBP, DBP, MBP, heart rate, respiratory rate, temperature SpO2, white blood cell count, hemoglobin, platelet count, anion gap, bicarbonate, sodium, potassium, blood urea nitrogen, calcium, chloride, creatinine, prothrombin time, glucose and triglycerides, with the exception of the subgroup variable.

To explore potential clinical heterogeneity, we conducted interaction and stratified analyses (Figure 5). We assessed the relationships between the TyG-BMI and short-term mortality across different subgroups stratified by age (<65 years and ≥ 65 years), sex, ethnicity, history of myocardial infarction, congestive heart failure, cerebrovascular disease, chronic pulmonary disease, diabetes, CRRT and septic shock. Significant interaction effects were observed only within the CRRT subgroup (*p* < 0.05) for the 28-, 90-, and 180-day mortality rates. No significant interactions were found in the other subgroups. The results showed that a higher TyG-BMI value was associated with a reduced risk of short-term death in the non-CRRT group.

### Sensitivity analysis

The results of the sensitivity analysis are presented in Table 3. After excluding patients with myocardial infarction, the hazard ratios (HRs) for 28-, 90-, and 180-day mortality were 0.998 (95% CI: 0.997–0.999), respectively. When patients with congestive heart failure or myocardial infarction were excluded, the HRs were 0.998 (95% CI: 0.997–0.999) for 28-day, 90-day, and 180-day mortality, respectively. Additionally, after patients with an ICU stay of less than 2 days were excluded, the HRs remained consistent at 0.998 (95% CI: 0.997–0.999), for 28-, 90-, and 180-day mortality, respectively. The trend test across the T1, T2, and T3 strata revealed statistical significance (*p* < 0.0001).

**Table 3:** Sensitivity analyses.

TyG-BMI	28-day mortality			90-day mortality			180-day mortality		
	HR (95%CI)	*p*	*p* for trend	HR (95%CI)	*p*	*p* for trend	HR (95%CI)	*p*	*p* for trend
Excluding participants with myocardial infarction									
Model	0.998 (0.997,0.999)	0.001		0.998 (0.997,0.999)	<0.0001		0.998 (0.997,0.999)	<0.0001	
Tertile1	Ref			Ref			Ref		
Tertile2	0.670 (0.545,0.823)	<0.0001		0.693 (0.579,0.829)	<0.0001		0.697 (0.587,0.828)	<0.0001	
Tertile3	0.673 (0.539,0.840)	<0.0001	<0.0001	0.649 (0.533,0.790)	<0.0001	<0.0001	0.654 (0.542,0.790)	<0.0001	<0.0001
Excluding participants with myocardial infarction and congestive heart failure									
Model	0.998 (0.997,0.999)	0.024		0.998 (0.997,0.999)	0.008		0.998 (0.997,0.999)	0.001	
Tertile1	Ref			Ref			Ref		
Tertile2	0.704 (0.554,0.895)	0.004		0.743 (0.604,0.913)	0.005		0.762 (0.626,0.927)	0.007	
Tertile3	0.717 (0.558,0.902)	0.009	<0.0001	0.696 (0.558,0.869)	<0.0001	<0.0001	0.712 (0.577,0.880)	<0.0001	<0.0001
Exclude all individuals with LOS ICU less than 2 days									
Model	0.998 (0.997,0.999)	<0.0001		0.998 (0.997,0.999)	<0.0001		0.998 (0.997,0.999)	<0.0001	
Tertile1	Ref			Ref			Ref		
Tertile2	0.690 (0.570,0.837)	<0.0001		0.695 (0.587,0.822)	<0.0001		0.686 (0.584,0.806)	<0.0001	
Tertile3	0.655 (0.529,0.811)	<0.0001	<0.0001	0.659 (0.547,0.795)	<0.0001	<0.0001	0.651 (0.544,0.778)	<0.0001	<0.0001

HR, hazard ratio; CI, confidence interval; Ref, reference; Adjusted for age, ethnicity, cerebrovascular disease, Charlson comorbidity index, SOFA score, SAPSII score, Septic shock, invasive ventilation, CRRT, SBP, DBP, MBP, Heart rate, Respiratory rate, Temperature SpO2, white blood cell count, hemoglobin, platelet count, anion gap, bicarbonate, sodium, potassium, blood urea nitrogen, calcium, chloride, creatinine, prothrombin time, glucose and Triglycerides.

## Discussion

This study, which was based on the MIMIC-IV database, analyzed the relationship between the TyG-BMI and short-term mortality in ICU patients with SA-AKI. The results revealed a significant L-shaped nonlinear relationship between the TyG-BMI and short-term all-cause mortality, particularly indicating that patients with lower TyG-BMIs faced a greater risk of death, with a critical threshold of 261.7. This relationship remained significant even after adjusting for multiple confounding factors, and sensitivity analyses further confirmed its stability across different subgroups, indicating that the TyG-BMI is an independent predictor of mortality. This association may be related to the “obesity paradox,” providing new insights for risk assessment and prevention strategies in patients with SA-AKI.

Previous studies have focused primarily on the relationship between TyG-BMI and cardiovascular events; for example, Dou et al. were the first to report its negative impact on all-cause mortality within 360 days in heart failure patients (24–27). While the prognostic value of the TyG-BMI for cardiovascular adverse outcomes, particularly all-cause mortality, has been confirmed (28–31), research on its relationship with SA-AKI is still limited. Notably, Fang et al. found that the TyG index was significantly associated with an increased risk of SA-AKI and prolonged hospital stays in septic patients (32). Additionally, Lou et al. reported that higher TyG index levels were associated with an increased risk of both hospital and ICU mortality in critically ill septic patients (33). These findings underscore the need for further research on how TyG-BMI affects the prognosis of patients with sepsis and SA-AKI, as such insights could provide valuable guidance for improving patient management in this specific population. This study specifically investigated the relationship between TyG-BMI and mortality in patients with SA-AKI. The results revealed that patients in the higher TyG-BMI group (T3) exhibited lower short-term mortality, despite potentially having more health issues. Kaplan–Meier survival analysis further confirmed that patients with lower TyG-BMIs (T1 group) had significantly higher mortality rates. The study revealed a nonlinear relationship between TyG-BMI and mortality: when the TyG-BMI was less than 261.7, the risk of death increased sharply, whereas above this threshold, the risk gradually decreased. Boruta analysis indicated that the TyG-BMI is an important predictor of short-term all-cause mortality.

In subgroup analyses, a significant interaction was observed only in the CRRT group (*p* < 0.05). The results indicate that in the non-CRRT group, higher TyG-BMI values are associated with a reduced risk of short-term mortality. These findings underscore the potential of the TyG-BMI as a prognostic indicator and individualized treatment tool. Future research should further explore the applications and mechanisms of the TyG-BMI in high-risk populations, providing more targeted guidance for clinical treatment decisions.

Most studies indicate that an elevated TyG index is associated with increased hospital and ICU mortality in critically ill patients (34–36). In ICU patients with sepsis, the TyG index has a U-shaped relationship with all-cause mortality (37, 38). The relationship between BMI and sepsis has also been well established, with individuals with lower BMIs exhibiting higher risks of hospitalization and all-cause mortality in sepsis patients, whereas overweight patients have lower risks (39–41). Our findings indicate that the TyG-BMI is associated with short-term all-cause mortality in SA-AKI patients in an L-shaped nonlinear manner, suggesting that the TyG-BMI may be a protective factor.

The correlation between the TyG-BMI and all-cause mortality in patients with SA-AKI may be influenced by both BMI and IR, a phenomenon known as the “obesity paradox” (42). Several mechanisms may explain this paradox: Patients with sepsis-associated acute kidney injury are often in a catabolic state, and a low TyG-BMI may reflect their malnutrition, resulting in insufficient metabolic reserves to cope with acute stressors such as sepsis. Additionally, studies have shown that low BMI is associated with poor prognosis in septic patients (42, 43), which could partly explain the higher mortality risk in patients with low TyG-BMI. A higher BMI or obesity may indicate greater physiological reserves, potentially leading to better outcomes (43). Obesity is associated with elevated glucose and fatty acid levels in high metabolic states, which may activate immune responses and affect inflammatory reactions, thereby improving disease outcomes (44, 45). Additionally, obese individuals typically have lower levels of B-type natriuretic peptide (BNP), indicating better hemodynamic characteristics, allowing them to better tolerate beneficial medications (46, 47). Finally, anti-inflammatory adipokines may play a protective role in obese patients.

This study suggests that the association between TyG-BMI and short-term mortality in SA-AKI may primarily be influenced by BMI, which could provide valuable guidance for clinical practice, although further validation is needed. TyG-BMI could serve as a predictive indicator for SA-AKI patients, particularly for those with low TyG-BMI, who are at higher risk of mortality and require close monitoring and early intervention. However, the relationship between TyG-BMI and mortality warrants further investigation. However, there are several limitations to this study. The retrospective design limits the establishment of causal relationships; although multivariable adjustments and subgroup analyses were conducted, potential confounding factors may still affect the results. The retrospective nature of the study may introduce selection bias and residual confounding factors, impacting its external validity. Additionally, since we only collected patient data within the first 24 h of admission, including indicators such as fasting glucose, triglycerides, weight, and height, the missing TyG-BMI data could impact the results. Furthermore, the study primarily assessed baseline TyG-BMI and did not capture dynamic changes in insulin resistance. Finally, research based on single-center data needs to be validated through multicenter studies to ensure the generalizability of the results. Future research should focus on broader samples and more rigorous designs to provide stronger evidence supporting the use of the TyG-BMI as a predictive indicator.

## Conclusion

In summary, this cohort study demonstrated that a higher TyG-BMI is strongly associated with reduced all-cause mortality in patients with SA-AKI. These findings suggest that the TyG-BMI may serve as a potential marker for early risk assessment, but further research is needed for validate.

## Data Availability

The original contributions presented in the study are included in the article/Supplementary material, further inquiries can be directed to the corresponding author.
