# Supplementary material for: L-shaped association between triglyceride-glucose body mass index and short-term mortality in ICU patients with sepsis-associated acute kidney injury
Source: Front Med (Lausanne). 2024 Dec 6;11:1500995. doi: 10.3389/fmed.2024.1500995 (PMC11659220; doi:10.3389/fmed.2024.1500995)
Supplement: Supplementary file 1 [file Table_1.docx]

**Table S1: Association between the TyG-BMI and all-cause mortality in SA-AKI after adjusting for BMI covariate in different models.**

| TyG-BMI | Model1  HR (95% CI) P value | | Model2  HR (95% CI) P value | | Model3  HR (95% CI) P value | | Model4  HR (95% CI) P value | |
| --- | --- | --- | --- | --- | --- | --- | --- | --- |
| 28-day mortality | 1.004 (1.001,1.008) | 0.019 | 0.999 (0.996,1.002) | 0.578 | 0.998 (0.995,1.002) | 0.295 | 0.990 (0.982,0.998) | 0.019 |
| Tertile1 | Ref | | Ref | | Ref | | Ref | |
| Tertile2 | 0.651(0.544,0.778) | <0.0001 | 0.641(0.523,0.785) | <0.0001 | 0.674(0.550,0.825) | <0.0001 | 0.699(0.565,0.864) | 0.001 |
| Tertile3 | 0.729(0.613,0.867) | <0.0001 | 0.714(0.530,0.961) | 0.026 | 0.704(0.522,0.949) | 0.021 | 0.757(0.542,0.1.056) | 0.101 |
| P for trend | <0.0001 | | <0.0001 | | <0.0001 | | <0.0001 | |
| 90-day mortality | 1.004 (1.001,1.007) | 0.022 | 0.999 (0.996,1.002) | 0.472 | 0.998 (0.995,1.001) | 0.017 | 0.994 (0.986,0.999) | 0.002 |
| Tertile1 | Ref | | Ref | | Ref | | Ref | |
| Tertile2 | 0.719(0.603,0.857) | <0.0001 | 0.651(0.556,0.762) | <0.0001 | 0.693(0.580,0.828) | <0.0001 | 0.712(0.591,0.858) | <0.0001 |
| Tertile3 | 0.855(0.658,1.111) | 0.242 | 0.663(0.565,0.777) | <0.0001 | 0.656(0.503,0.855) | 0.002 | 0.703(0.524,0.944) | 0.0019 |
| P for trend | <0.0001 | | <0.0001 | | <0.0001 | | <0.0001 | |
| 180-day mortality | 1.003 (1.001,1.006) | 0.06 | 0.998 (0.995,1.002) | 0.287 | 0.997 (0.994,1.000) | 0.078 | 0.993 (0.986,1.000) | 0.024 |
| Tertile1 | Ref | | Ref | | Ref | | Ref | |
| Tertile2 | 0.713(0.603,0.844) | <0.0001 | 0.647(0.546,0.766) | <0.0001 | 0.693(0.585,0.821) | <0.0001 | 0.716(0.599,0.856) | <0.0001 |
| Tertile3 | 0.837(0.651,1.077) | 0.166 | 0.645(0.500,0.832) | 0.001 | 0.648(0.502,0.837) | 0.001 | 0.708(0.533,0.941) | 0.017 |
| P for trend | <0.0001 | | <0.0001 | | <0.0001 | | <0.0001 | |

HR, hazard ratio; CI, confidence interval;

Model 1: Adjusted for BMI.

Model 2: Adjusted for variables included in Model 1+ age, ethnicity, cerebrovascular disease, the Charlson comorbidity index, SOFA score, SAPSII score, septic shock, invasive ventilation and CRRT.

Model 3: Adjusted for variables included in Model 2 + SBP, DBP, MAP, heart rate, respiratory rate, temperature and SpO2.

Model 4: Adjusted for variables included in Model 3 + white blood cell count, hemoglobin, platelet count, anion gap, bicarbonate, calcium, blood urea nitrogen, sodium, potassium, chloride, creatinine, prothrombin time, glucose and Triglycerides.
