# Supplementary material for: L-shaped association between triglyceride-glucose body mass index and short-term mortality in ICU patients with sepsis-associated acute kidney injury
Source: Front Med (Lausanne). 2024 Dec 6;11:1500995. doi: 10.3389/fmed.2024.1500995 (PMC11659220; doi:10.3389/fmed.2024.1500995)
Supplement: Supplementary file 2 [file Table_2.docx]

**Table S2: Association between the TyG-BMI and all-cause mortality in SA-AKI after adjusting for TyG covariate in different models.**

| TyG-BMI | Model1  HR (95% CI) P value | | Model2  HR (95% CI) P value | | Model3  HR (95% CI) P value | | Model4  HR (95% CI) P value | |
| --- | --- | --- | --- | --- | --- | --- | --- | --- |
| 28-day mortality | 0.998 (0.997,0.999) | <0.0001 | 0.998 (0.997,0.999) | <0.0001 | 0.998 (0.997,0.999) | 0.001 | 0.998 (0.997,0.999) | 0.004 |
| Tertile1 | Ref | | Ref | | Ref | | Ref | |
| Tertile2 | 0.601(0.500,0.722) | <0.0001 | 0.614(0.510,0.740) | <0.0001 | 0.650(0.540,0.783) | <0.0001 | 0.697(0.578,0.842) | <0.0001 |
| Tertile3 | 0.622(0.510,0.758) | <0.0001 | 0.650(0.532,0.794) | <0.0001 | 0.650(0.532,0.794) | <0.0001 | 0.718(0.585,0.881) | 0.002 |
| P for trend | <0.0001 | | <0.0001 | | <0.0001 | | <0.0001 | |
| 90-day mortality | 0.998 (0.997,0.999) | <0.0001 | 0.998 (0.997,0.999) | <0.0001 | 0.998 (0.997,0.999) | <0.0001 | 0.999 (0.998,1.000) | 0.004 |
| Tertile1 | Ref | | Ref | | Ref | | Ref | |
| Tertile2 | 0.627(0.534,0.735) | <0.0001 | 0.645(0.548,0.758) | <0.0001 | 0.694(0.590,0.816) | <0.0001 | 0.730(0.620,0.860) | <0.0001 |
| Tertile3 | 0.615(0.516,0.733) | <0.0001 | 0.650(0.544,0.776) | <0.0001 | 0.656(0.549,0.783) | <0.0001 | 0.714(0.596,0.856) | <0.0001 |
| P for trend | <0.0001 | | <0.0001 | | <0.0001 | | <0.0001 | |
| 180-day mortality | 0.998 (0.997,0.999) | <0.0001 | 0.998 (0.997,0.999) | <0.0001 | 0.998 (0.997,0.999) | <0.0001 | 0.998 (0.997,0.999) | 0.001 |
| Tertile1 | Ref | | Ref | | Ref | | Ref | |
| Tertile2 | 0.624(0.535,0.726) | <0.0001 | 0.645(0.553,0.753) | <0.0001 | 0.696(0.596,0.812) | <0.0001 | 0.729(0.624,0.852) | <0.0001 |
| Tertile3 | 0.605(0.512,0.716) | <0.0001 | 0.642(0.542,0.761) | <0.0001 | 0.651(0.549,0.771) | <0.0001 | 0.704(0.592,0.837) | <0.0001 |
| P for trend | <0.0001 | | <0.0001 | | <0.0001 | | <0.0001 | |

HR, hazard ratio; CI, confidence interval;

Model 1: Adjusted for TyG.

Model 2: Adjusted for variables included in Model 1+ age, ethnicity, cerebrovascular disease, the Charlson comorbidity index, SOFA score, SAPSII score, septic shock, invasive ventilation and CRRT.

Model 3: Adjusted for variables included in Model 2 + SBP, DBP, MAP, heart rate, respiratory rate, temperature and SpO2.

Model 4: Adjusted for variables included in Model 3 + white blood cell count, hemoglobin, platelet count, anion gap, bicarbonate, calcium, blood urea nitrogen, sodium, potassium, chloride, creatinine, prothrombin time, glucose and Triglycerides.
